# MiR-497 decreases cisplatin resistance in ovarian cancer cells by targeting mTOR/P70S6K1

**DOI:** 10.18632/oncotarget.4762

**Published:** 2015-07-03

**Authors:** Shaohua Xu, Guang-Bo Fu, Zhen Tao, Jun OuYang, Fanfei Kong, Bing-Hua Jiang, Xiaoping Wan, Ke Chen

**Affiliations:** ^1^ Department of Obstetrics and Gynecology, Shanghai First Matenity and Infant Hospital, Tongji University School of Medicine, Shanghai, China; ^2^ Department of Urology and Pathology, Huai'an First People's Hospital, Nanjing Medical University, Huai'an, China; ^3^ Cancer Center, Union Hospital, Tongji Medical College, Huazhong University of Science and Technology, Wuhan, China; ^4^ Department of Science and Technology, Radiation Oncology Department, Tianjin Medical University Cancer Hospital and Institute, Tianjin, China; ^5^ Changzhou Maternal and Child Health Hospital Affiliated to Nanjing Medical University, Changzhou, China; ^6^ State Key lab of Reproductive Medicine, Cancer Center, Nanjing Medical University, China; ^7^ Department of Pathology, Anatomy and Cell Biology, Thomas Jefferson University, Philadelphia, PA, USA

**Keywords:** miR-497, mTOR, p70S6K1, cisplatin resistance, ovarian cancer

## Abstract

The mechanism of cisplatin resistance in ovarian cancer is not clearly understood. In the present investigation, we found that the expression levels of miR-497 were reduced in chemotherapy-resistant ovarian cancer cells and tumor tissues due to hypermethylation of miR-497 promoter. Low miR-497 expression levels were associated with chemo-resistant phonotype of ovarian cancer. By analyzing the expression levels of miR-497, mTOR and p70S6K1 in a clinical gene-expression array dataset, we found that mTOR and p70S6K1, two proteins correlated to chemotherapy-resistance in multiple types of human cancers, were inversely correlated with miR-497 levels in ovarian cancer tissues. By using an orthotopic ovarian tumor model and a Tet-On inducible miR-497 expression system, our results demonstrated that overexpression of miR-497 sensitizes the resistant ovarian tumor to cisplatin treatment. Therefore, we suggest that miR-497 might be used as a therapeutic supplement to increase ovarian cancer treatment response to cisplatin.

## INTRODUCTION

Epithelial ovarian carcinoma is the leading cause of death worldwide from gynecological malignancies [1]. Despite current multidisciplinary treatment, the overall prognosis remains disappointing [2, 3]. More than 75% of patients diagnosed with epithelial ovarian carcinoma are at advanced-staged, and the 5-year survival rate is less than 30% [4]. Cisplatin is one of the most effective and commonly used chemotherapeutics agents for the treatment of ovarian cancer. However, the development of cisplatin-based resistance limits its successful clinical application in cancer patients [5]. Despite the knowledge that has been accumulated over decades, the mechanisms of cisplatin resistance are not fully understood.

MiRNAs are small endogenous non-coding RNAs composed of about 19-24 nucleotides that bind to imperfect sequence homology sites of mRNA and recruit the RNA-induced silencing complex, causing either degradation or inhibition of protein translation, thus effectively silencing their mRNA targets [6]. It has been reported that miRNA-mediated gene regulation involvoed in biological processes including cell proliferation, migration and invasion, differentiation, survival, and tumorigenesis [7, 8]. Recent studies showed that miRNAs also play a vital role in chemotherapeutic resistance [9, 10], highlighting miRNAs as potent therapeutic targets or chemoresistant modulators in cancer treatment. MiR-497 is one of the tumor suppressor miRNAs in human cancer. Downregulation of miR-497 has been found in breast, cervical, head-and-neck, colorectal, and prostate cancer [11–14]. Reduced expression of miR-497 has been associated with malignancy of breast and colorectal cancer [11, 14]. In addition, forced expression of miR-497 is able to suppress cancer cell growth both *in vitro* and *in vivo* [15–16]. Finally, increasing evidence indicates that miR-497 negatively regulates numerous well-characterized oncogenic proteins, such as IGF-1R [13], CCND1 [11], CDC25A, CDK6, CDK4 [17], and BCL-2 [18]. Although miR-497 has been shown to be a tumor suppressor gene in many human cancers, its role in chemotherapeutical resistance has not been fully addressed. The objective of this study was to reveal the molecular mechanisms of miR-497 in cisplatin-resistant ovarian cancer.

## RESULTS

### MiR-497 expression was downregulated in cisplatin-resistant ovarian cancer cell lines and ovarian cancer specimens

**Figure 1:**
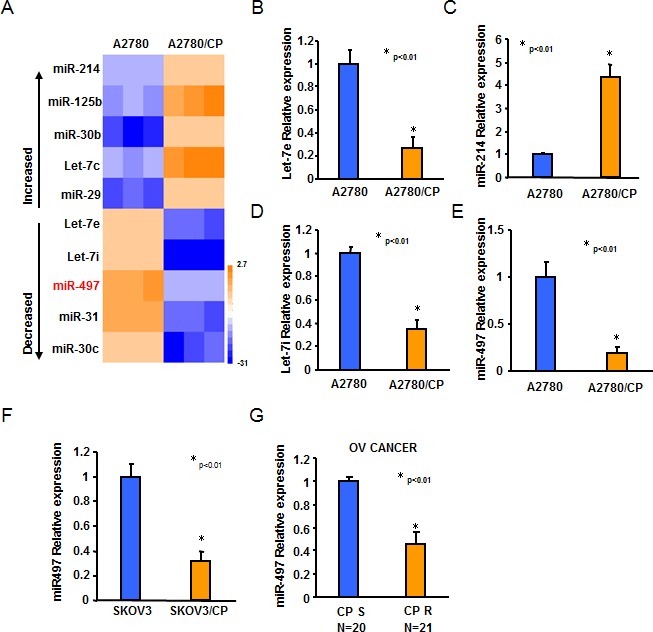
The expression levels of miR-497 were downregulated in cisplatin-resistant ovarian cancer cells **A.** miRNA array analysis showed that miRNAs were differentially expressed in A2780 and A2780/CP cells. The pseudocolar represents the intensity scale of A2780 versus A2780/CP cells. **B.**-**E.** Relative expression levels of Let-7e, Let-7i, miR-214, and miR-497 in A2780 and A2780/CP cells were determined by Taqman qRT-PCR assay, and normalized to the U6 levels. **F.** Relative expression levels of miR-497 in SKOV3 and SKOV3/CP cells were determined by Taqman qRT-PCR assay, and normalized to the U6 levels. **G.** Relative expression levels of miR-497 in 20 different platinum-sensitive and 21 different platinum-resistant ovarian tumors. All results represent the mean ± SD from three independent experiments.

To determine the crucial miRNAs involved in ovarian cancer cisplatin resistance, we performed microarray assay to profile the global expression of mature miRNAs in A2780 and A2780/CP cell lines. The signal ratios of A2780/CP to A2780 were assessed. Differentially expressed miRNAs with at least 2-fold alternation were selected (Figure 1A). Consistent with other studies, we also found that let-7e and let-7i were among the top 10 downregulated miRNAs and miR-214 was upregulated in A2780/CP compared with A2780. Importantly, miR-497 was remarkably downregulated in A2780/CP compared with A2780 (Figure 1B–1E). To further validate this finding, we examined miR-497 expression levels in SKOV3 and SKOV3/CP cell lines. The results showed that miR-497 levels were significantly reduced in SKOV3/CP cells compared with SKOV3 cells (Figure 1F). We further investigated the association of miR-497 levels in primary ovarian tumors and its response to platinum-based chemotherapy. We found that miR-497 levels were significant lower in platinum sensitive tumors compared with platinum resistant tumors (Figure 1G), indicating that miR-497 may play an important role in the development of cisplatin resistance in ovarian cancer.

### MiR-497 downregulation was due to DNA methylation

**Figure 2:**
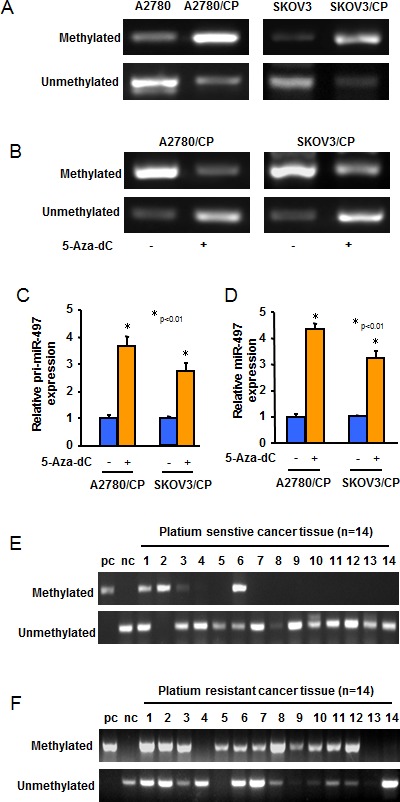
The expression of miR-497 was regulated by DNA methylation **A.** MSP analyses of *miR-497* gene promoter in A2780, A2780/CP, SKOV3 and SKOV3/CP cells. U indicated unmethylated status; M indicated methylated status. **B.** A2780/CP and SKOV3/CP cells were treated with 5-Aza-dC for 5 days. The methylation of miR-497 promoter in the cells was analyzed using MSP. **C.**-**D.** A2780/CP and SKOV3/CP cells were treated without or with 5-Aza-dC for 5 days. Pri-miR-497 and miR-497 expression levels were measured by qRT-PCR. The graphs show the mean ± SD of the relative levels from three replications. **E.**-**F.** MSP analyses of *miR-497* gene promoter in 14 different pairs of platinum-sensitive and platinum-resistant ovarian tumors.

To explore the mechanism of miR-497 downregulation in cisplatin-resistant ovarian cancer cells, we first analyzed the genomic DNA sequence within 3-kilobase promoter regions of miR-497 gene, and found miR-497 gene contains CpG-rich regions (CpG islands) in its promoter regions. We compared methylation status of the promoter of miR-497 in A2780 and A2780/CP or in SKOV3 and SKOV3/CP cells by methylation-specific PCR (MSP) analysis. Hypermethylation of miR-497 promoter was identified in A2780 and SKOV3 cells compared with A2780/CP and SKOV3/CP cells, respectively (Figure 2A–2B). To further determine whether DNA methylation is responsible for miR-497 downregulation, we treated A2780/CP and SKOV3/CP cells with or without 5-Aza-dC, a demethylation reagent, and performed MSP assay. Demethylation treatment by 5-Aza-dC dramatically restored both pri-miR-497 and matured miR-497 expression levels in A2780/CP and SKOV3/CP cells (Figure 2C–2D), indicating that hypermethylation plays a crucial role in the silencing of miR-497 expression. We next analyzed miR-497 promoter methylation status in 28 ovarian cancer samples. The MSP results showed that the methylation levels of miR-497 promoter regions in platinum resistant tumors were dramatically higher than those in platinum sensitive tumors (Figure 2E–2F). Collectively, these results indicated that DNA hypermethylation may be the main reason for miR-497 downregulation in ovarian cancer cells.

### MiR-497 is involved in cisplatin-resistant ovarian cancer phonotype

**Figure 3:**
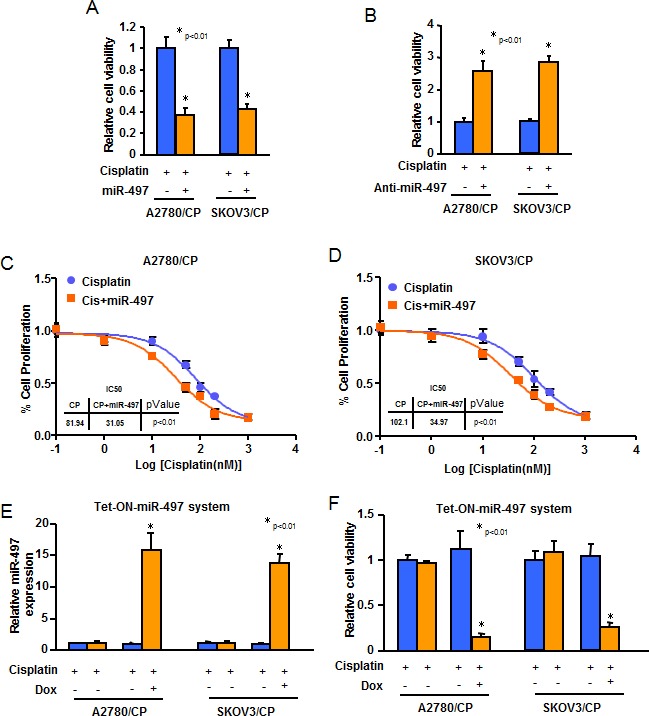
Overexpression of miR-497 reduces resistance of ovarian cancer cells to cisplatin treatment **A.**-**B.** A2780/CP and SKOV3/CP cells were transfected with pre-miR-497, or scrambled miRNA precursors **A.** or with anti-miR-497, or scrambled miRNA inhibitors **B.**. Cells were treated with cisplatin for 72 h. The cell viability rate was analyzed by MTT assay. **C.**-**D.** Serum starved A2780/CP and SKOV3/CP cells with stable overexpression of miR-497, then exposed to various concentrations of cisplatin for 48 h. IC_50_ values were determined. **E.** A2780/CP and SKOV3/CP cells were transfected with pCMV-Tet3G, and selected by G418 for stably transfected cells. Then cells were transfected with pTRE3G-miR-497 or pTRE3G-miR-NS and were selected by puromycin. Cells were exposed to Doxycycline (Dox) (500 ng/ml) for 48 h, and total RNAs were extracted. The expression levels of miR-497 were analyzed by Taqman qRT-PCR assay. **F.** Cells as indicated were exposed to Dox (500 ng/ml) for 48 h before treated with cisplatin for 72 h. MTT assay was used to test cell viability after cisplatin treatment. All results represent the mean ± SD from three independent experiments.

To investigate the roles of miR-497 in cisplatin-resistant phonotype of ovarian cancer cells, we forced expression of miR-497 in A2780/CP and SKOV3/CP cells with low endogenous miR-497 levels by transfection of cells with miR-497 precursor or a control precursor (miR-NS). We also inhibited levels of miR-497 in A2780 and SKOV3 cells by transfection of cells with anti-miR-497 inhibitor or a control inhibitor (anti-miR-NS). MiR-497 overexpression dramatically reduced A2780/CP and SKOV3/CP cells resistance to cisplatin (Figure 3A). On the contrary, inhibition of miR-497 expression markedly increased A2780 and SKOV3 cells tolerance to cisplatin (Figure 3B). Furthermore, consistent with the results from MTT assay, we found that overexpression of miR-497 significantly sensitized A2780/CP and SKOV3/CP cells to cisplatin treatment (Figure 3C–3D). Next, we generated Tet-On-based stable cell lines for inducible expression of miR-497 in A2780/CP-Tet-ON-miR-497 and SKOV3/CP-Tet-ON-miR-497 cells. Doxycycline (Dox) treatment significantly increased miR-497 expression in A2780/CP-Tet-ON-miR-497 and SKOV3/CP-Tet-ON-miR-497 cells, as compared with miR-NS expressing cells or doxycycline non-treatment groups (Figure 3E). We observed that miR-497 over-expression in A2780/CP and SKOV3/CP cells resulted in much stronger cisplatin treatment response than transient miR-497 restoration. Dox treatment altered miR-497 expression or cisplatin resistance of both A2780/CP and SKOV3/CP cells (Figure 3F). Taken together, downregulation of miR-497 expression render ovarian cancer cells resistant to cisplatin treatment.

### mTOR and p70S6K1 are direct targets of miR-497

**Figure 4:**
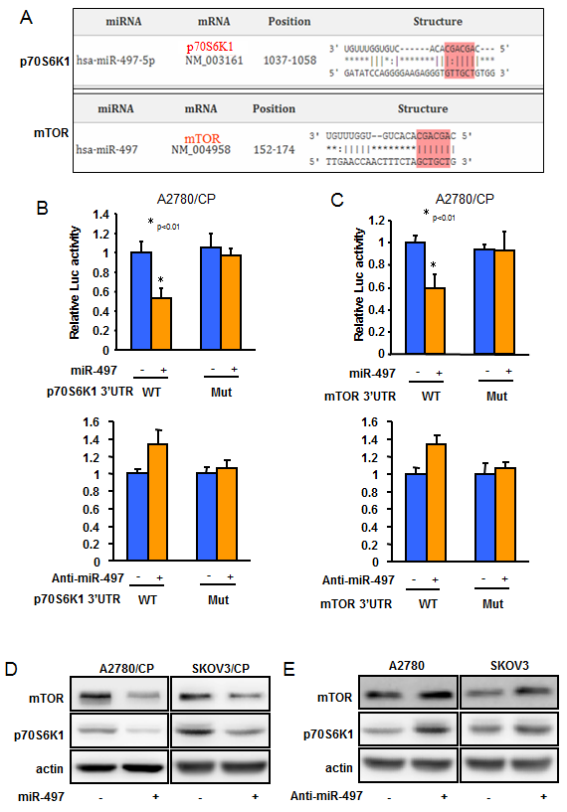
mTOR and p70S6K1 are two direct targets of miR-497 **A.** The figure shows the alignment of miR-497 putative binding sites in human mTOR and p70S6K1 3′-UTR regions. **B.**-**C.** The reporter constructs containing the wild-type and mutant (Mut) p70S6K1 **B.** or mTOR **C.** 3′ -UTR regions were co-transfected into A2780/CP cells with miR-497, or scramble miRNA precursors; or anti-miR-497, or anti-scramble miRNA inhibitors and β-gal plasmid. The relative luciferase/β-gal activities were analyzed in the cells 48 h after the transfection. All experiments were performed in triplicate. Bars indicate relative luciferase activities ± SD. * indicated *p* < 0.05. **D.** A2780/CP and SKOV3/CP cells were transfected with miR-497 or miR-Scr; or **E.** A2780 and SKOV3 cells were transfected with anti-miR-497 or anti-miR-Scr inhibitors for 72 h. The expression levels of mTOR or p70S6K1 were analyzed by Western blotting. Relative expression levels of mTOR or p70S6K1 in 21 different platinum-sensitive and 20 different platinum-resistant ovarian tumors.

To investigate the molecular mechanisms of how miR-497 decreases cisplatin resistance, we employed several well-developed miRNA algorithms, such as TargetScan, PicTar, and miRNA.org, to obtain a list of possible mRNA targets of miR-497. Among the search results, mTOR and p70S6K1 captured our attention because they were reported to be involved in cisplatin resistance in multiple human cancers [19–21] (Figure 4A). To confirm these targets, reporter constructs were made to contain the putative binding sites of mTOR and p70S6K1 3′-UTR regions, or with 3 nucleotide substitute in their 3′-UTR regions (Mut). The luciferase activities from the mTOR and p70S6K1 wild-type construct were inhibited upon overexpression of miR-497 and were induced by inhibition of miR-497. Point mutations in putative binding site abrogated the effect of miR-497, demonstrating that miR-497 specifically target the mTOR and p70S6K1 3′ UTR by binding to the identified seed sequence (Figure 4B–4C). Furthermore, forced expression of miR-497 by transient transfection repressed both mTOR and p70S6K1 protein expression in A2780/CP and SKOV3/CP cells; whereas blockade of endogenous miR-497 using antisense inhibitors increased both mTOR and p70S6K1 expression levels in A2780 and SKOV3 (Figure 4D–4E). Similar effects of miR-497 on targeting mTOR and p70S6K1 have also been observed in multiple human cancer cells (data not shown). No significant differences were found in mTOR and p70S6K1 mRNA levels (data not shown), indicating that mTOR and p70S6K1 expression is regulated at the translational level through miR-497. These results demonstrated that miR-497 directly targets mTOR and p70S6K1 to repress their protein expression by binding to their 3′-UTR regions.

### MiR-497 decreases cisplatin resistance through targeting mTOR and p70S6K1

**Figure 5:**
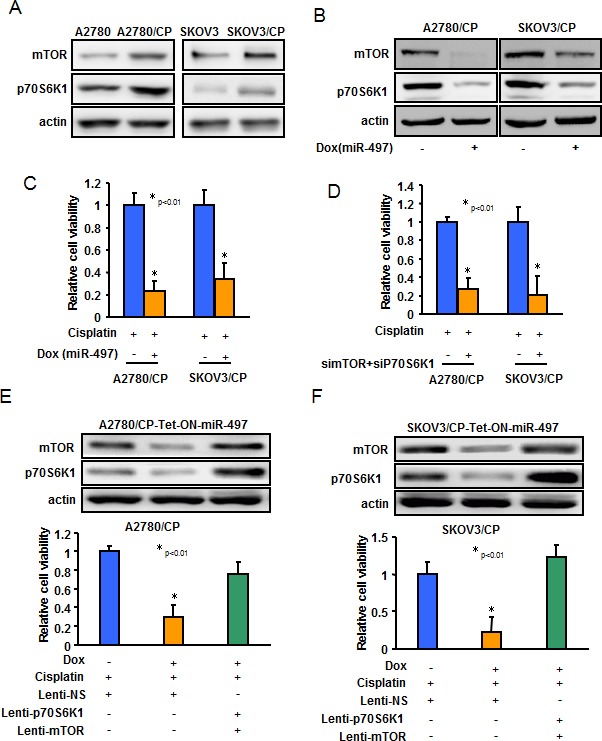
MiR-497 inhibits cisplatin resistance through targeting mTOR and p70S6K1 in ovarian cancer cells **A.**-**B.** The expression levels of mTOR and p70S6K1 in A2780, A2780/CP, SKOV3 and SKOV3/CP cells **A.**; or in A2780/CP-Tet-ON-miR-497 or SKOV3/CP-Tet-ON-miR-497 cells exposed to Dox for 48 h **B.**, were measured by Western blotting. **C.** A2780/CP-Tet-ON-miR-497 or SKOV3/CP-Tet-ON-miR-497 cells were exposed to Dox for 48 h and treated with cisplatin for 72 h. The cells viability rate was analyzed by MTT assay. **D.** A2780/CP and SKOV3/CP cells were transfected with si-mTOR or si-p70S6K1, or scrambled control. Cells were treated with cisplatin for 72 h. The cells viability rate was analyzed by MTT assay. **E.**-**H.** A2780/CP-Tet-ON-miR-497 or SKOV3/CP-Tet-ON-miR-497 cells were maintained in the medium with or without Dox and were infected using lentivirus carrying Scr, mTOR or p70S6K1 for 48 h. Then the cells were treated with cisplatin for 72 h before MTT assay.

We found that the expression levels of mTOR and p70S6K1 were upregulated in cisplatin-resistant ovarian cancer cells (A2780/CP and SKOV3/CP) compared with cisplatin-sensitive cells (A2780 and SKOV3) (Figure 5A). To investigate whether miR-497 overexpression enhanced the cisplatin response sensitivity of ovarian cancer cells *via* targeting mTOR and p70S6K1, we performed mTOR and p70S6K1 loss- and gain-of-function experiments in ovarian cancer cells. Doxycycline treatment-induced stable-expressing miR-497 constantly decreased both mTOR and p70S6K1 expression in A2780/CP and SKOV3/CP, as well as dramatically reduced cells resistance to cisplatin treatment (Figure 5B–5C). Knockdown of endogenous mTOR and p70S6K1 exerted a similar effect as overexpression of miR-497 on decreasing resistance of ovarian cancer cells (Figure 5D). Next, we used lentiviral particles carrying mTOR or p70S6K1 cDNAs lacking their 3′-UTR regions to infect miR-497 stable-expressing ovarian cancer cells. Interestingly, forced expression of mTOR and p70S6K1 partially or completely restored miR-497-inhibited cisplatin resistance in ovarian cancer cells (Figure 5E–5F). These results indicated that miR-497 downregulation promotes acquisition of cisplatin-resistant ability in ovarian cancer cell *via* inducing mTOR and p70S6K1 overexpression.

### MiR-497 attenuates cisplatin resistance in orthotopic ovarian animal model

**Figure 6:**
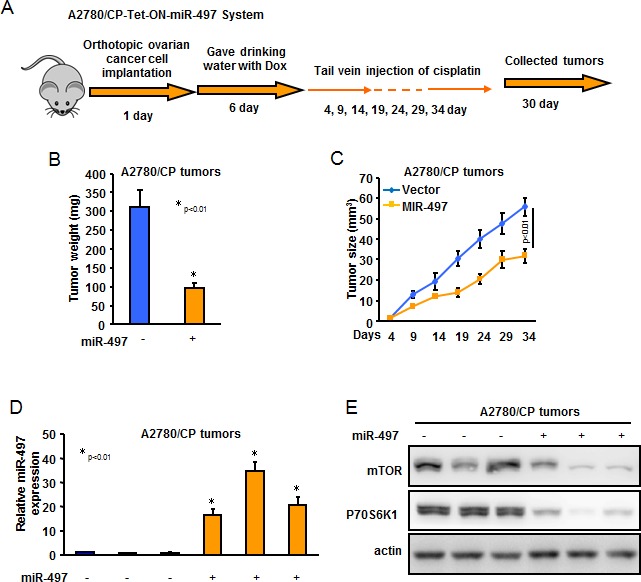
MiR-497 overexpression decreased cisplatin resistance of ovarian cancer cells *in vivo* **A.** Schematic of animal experimental procedure. **B.** Tumor weight from two groups was obtained and presented. **C.** Tumor size was measured every 5 days and tumor growth curves were showed. **D.** The expression levels of miR-497 in tumor tissues were analyzed by qRT-PCR. **E.** The representative image of the protein levels of mTOR and p70S6K1 were analyzed by western blotting.

To test whether restoration of miR-497 expression in cisplatin-resistant ovarian cancer cells can enhance the response of cisplatin treatment *in vivo*, we established an orthotopic ovarian tumor model by implantation of A2780/CP-Tet-ON-miR-497 or A2780/CP-Tet-ON-miR-NS cells in nude mouse ovary. On Day 6 after cell implantation, tumor-bearing mice were given drinking water with Dox every day to induce miR-497 or miR-NS expression. On Day 4, the mice received tail vein injection of cisplatin every five days for a total of 30 days. After 34 days of cell implantation, the mice were euthanized and tumors were collected (Figure 6A). As shown in Figure 6B–6D, tumor weights and volume in the miR-497 + cisplatin group were smaller than those in the miR-NS + cisplatin group. The levels of miR-497 were significantly higher and the levels of mTOR and p70S6K1 were dramatically lower in A2780/CP-Tet-ON-miR-497 formed tumors than those in A2780/CP-Tet-ON-miR-NS formed tumors (Figure 6E–6F).

### MiR-497 levels were inversely correlated with mTOR and p70S6K1 levels in tissues from ovarian cancer patients

**Figure 7:**
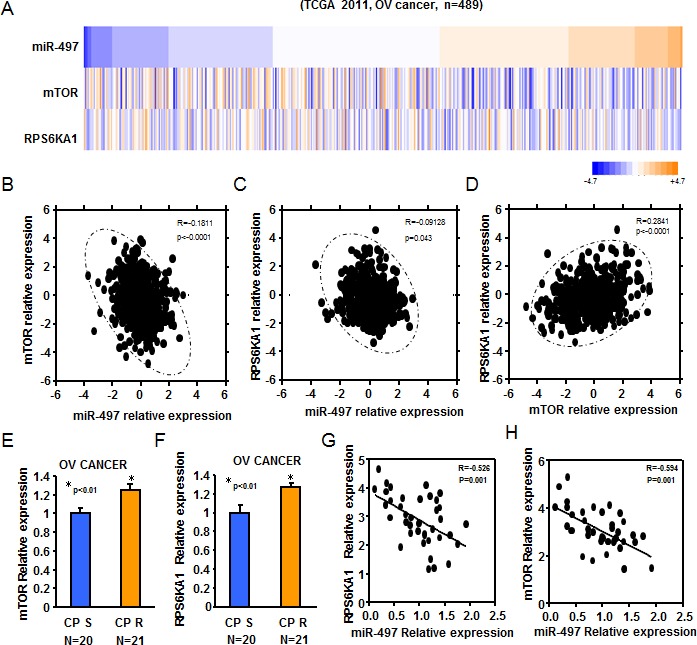
Reduced expression of miR-497 was inversely correlated to mTOR/p70S6K1 levels of ovarian cancer patients **A.** Heatmap depicting data from TCGA 2011 ovarian cancer microarray datasets that were assigned to ovarian cancer gene expression subtypes (*n* = 489). **B.** miR-497 levels were inversely correlated to mTOR expression levels in human ovarian cancer (*R* = −0.1811, *P* < 0.0001). **C.** miR-497 levels were inversely correlated to p70S6K1 levels in human ovarian cancer (*R* = −0.09128, *P* = 0.043). **D.** mTOR expression levels were positively correlated to p70S6K1 levels in human ovarian cancer (*R* = −0.2841, *P* < 0.0001). **E.**-**F.** Real-time PCR was also performed to determine mTOR and p70S6K1 expression in 20 different platinum-sensitive and 21 different platinum-resistant ovarian tumors. Both mTOR and p70S6K1 levels were significantly upregulated in platinum resistant tumors compared with platinum sensitivity tumors. **G.**-**H.** Spearman's rank correlation analysis revealed an inverse correlation between miR-497 and mTOR levels (*R* = −0.526, *P* = 0.001), and p70S6K1(*R* = −0.594, *P* = 0.001) exists in our ovarian cancer tumor samples.

The above studies showed that miR-497 directly target mTOR and p70S6K1 to repress their protein expression by binding to their 3′-UTR regions. To further test the physiological relevance of mTOR/p70S6K1 and miR-497 interaction, we performed in silico analysis for miR-497 and mTOR/p70S6K1 expression from The Cancer Genome Atlas (TCGA) 2011 dataset for Ovarian Cancer, comprising of 489 patient samples with follow-up information (Figure 7A). As shown in Figure 7B–7D, miR-497 and mTOR/p70S6K1 were inversely correlated in ovarian cancer tissues, while mTOR and p70S6K1 were positively correlated.

Next, mTOR and p70S6K1 mRNA levels were quantified using real-time PCR. We observed that both mTOR and p70S6K1 were significantly upregulated in platinum resistant tumors compared with platinum sensitivity tumors, respectively (Figure 7E–7F). Furthermore, spearman's rank correlation analysis revealed an inverse correlation between miR-497 and mTOR, and p70S6K1 exists in our ovarian cancer samples (Figure 7G–7H). Taken together, these results showed lower expression levels of miR-497 were inversely correlated with mTOR/p70S6K1 levels of ovarian cancer patients.

## DISCUSSION

Previous studies have reported that miRNAs are aberrantly expressed in ovarian cancer and are associated with tumor stages, histological subtypes, recurrent tumors and survival [22–26]. Recently, increasing evidence also showed that miRNAs can affect sensitivity of ovarian cancer to various chemotherapy agents. For example, low levels of let-7i associated with chemoresistance in ovarian cancer and knockdown of let-7i decreased cisplatin-induced ovarian cancer cell death [10]. MiR-200c overexpression may increase the sensitivity to microtubule-targeting drugs through repressing the expression of the tubulin molecule TUBB3 [27]. In addition, miR-214 is involved in chemosensitivity by modulating PTEN expression, an important tumor suppressor gene [9]. Thus far, only a limited number of miRNAs has been found to impact chemoresistance in ovarian cancer and the effect of miRNAs in mediating sensitivity to anticancer treatment in ovarian cancer remain incompletely understood. In the present study, we performed miRNA expression profiling in cisplatin-sensitive cell lines (A2780) and cisplatin-resistant cell lines (A2780/CP). Our results showed that downregulation of let-7i, let-7e and upregulation of miR-214 in A2780/CP compared with A2780. Based on our previously studies [28, 29], we identified miR-497 as a new miRNA involved in cisplatin sensitivity. The expression levels of miR-497 were significantly decreased in both cisplatin-resistant cell lines (A2780/CP and SKOV3/CP) as well as in platinum-resistant ovarian cancer tissues. Overexpression of miR-497 by transient transfection or Tet-ON-induced system decreased cisplatin-resistance of A2780/CP and SKOV3/CP cells.

Epigenetic modification such as DNA methylation has been shown to play an important role in the development of cisplatin resistance. Demethylation drug treatments have been shown to reverse cisplatin resistance by restoring the expression of certain resistance-related gene expression [30, 31]. However, there is less evidence showing that epigenetic regulated miRNAs contribute to the development of a cisplatin-resistant phenotype. Our results demonstrated that increased methylation of the miR-497 promoter play a crucial role for silencing expression of miR-497 and demethylation by 5-AZA treatment can reverse miR-497 expression in cisplatin-resistant ovarian cancer cells. The molecular mechanism of cisplatin-induced hyper-methylation of miR-497 gene will be the focus of our future study.

Our results demonstrated that mTOR and p70S6K1 are two direct targets of miR-497, highlighting the potential role of miR-497 downregulation for ovarian cancer cells to develop a chemoresistance phenotype. The mTOR/p70S6K1 signaling pathway has been reported as a critical regulator of cellular metabolism, growth, survival, and drug resistance [32–34]. Overexpression of mTOR or p70S6K1 has been found in many human cancers and is associated with tumor malignancy and poor prognosis [35, 36]. However, the mechanisms of posttranscriptional regulation of mTOR or p70S6K1 have yet to be elucidated. We showed that miR-497 could suppress both mTOR and p70S6K1 protein expressions through the binding of these miRNAs to their 3′-UTR regions. Downregulation of miR-497 contributes to high levels of mTOR and p70S6K1, which makes ovarian cancer cells resistant to cisplatin-based chemotherapy.

To study the impact of miR-497 in ovarian cancer tumor resistance to cisplatin treatment *in vivo*, we employed an orthotopic ovarian tumor model and a Tet-On inducible miR-497 system. In the orthotopic ovarian tumor model, ovarian tumors were formed in mice normal ovary, which provides a better microenvironment than subcutaneous injection. The advantage of the Tet-On inducible miR-497 system is that the expression of miR-497 is more controllable by addition or depletion of Dox in drinking water than long-term continuous stable-expressing miR-497 cell lines. Our data showed that induced-expressing of miR-497 in cisplatin-resistant ovarian cancer cells exert a much better cisplatin treatment effect on ovarian tumor growth than control groups, indicating a possible application for miR-497 as a “supplement” to conventional cisplatin-based chemotherapy. Applied cisplatin with miR-497 may help to reduce the rate of cisplatin-resistant ovarian cancer occurrence and improve the overall response rate to chemotherapy.

In conclusion, our study demonstrated that 1) miR-497 was downregulated in cisplatin-resistant ovarian cancer cell lines and tumors; 2) DNA hypermethylation contribute to downregulation of miR-497; 3) mTOR and p70S6K1 are two direct targets of miR-497 with biological function that are associated with development of cisplatin-resistance phonotype; and 4) overexpression of miR-497 decreases cisplatin resistance of ovarian cancer cells *in vitro* and *in vivo*.

## MATERIALS AND METHODS

### Cell lines and reagents

The human ovarian cancer cell lines A2780, A2780/CP, SKOV3, and SKOV3/CP were purchased from The Cell Bank of Chinese Academy of Science. Cells were maintained in a medium of RPMI 1640 supplemented 10 % FBS and 1 % penicillin/streptomycin and were kept in 5 % CO_2_ incubator at 37°C. Antibodies against mTOR, p70S6K1 and β-actin were purchased from Cell Signaling Technology. siRNAs targeting mTOR or p70S6K1 were purchased from Thermo Scientific as specific oligo pools. For demethylation treatment, cells were treated with 5 μM of 5-Aza-2′-deoxycytidine (5-Aza-dC) (Sigma, MO, USA) for 120 h, with replacing the fresh drug each day. Cisplatin was obtained from Sigma (St. Louise, MO).

### Tissue samples

A collection of 41 different kinds of fresh-frozen ovarian cancer tumor specimens were from the tissue bank of Nanjing Medical University. All tumor samples were collected immediately after the surgical removal and snap-frozen in liquid nitrogen. The clinical characteristics of the ovarian cancer patients are listed in Table 1. All patients provided written informed consent and the experimental procedures were approved by the Institutional Review Board of the Nanjing Medical University. Progression-free survival (PFS) was calculated from time of surgery to time of progression or recurrence. PFS > 6 months was defined as sensitivity to the last platinum-based chemotherapy; and PFS < 6 months was defined as resistant to the last platinum-based chemotherapy.

**Table 1:** Clinical characteristics of ovarian cancer patients

Patient Characteristics (*N* = 41)		
Characteristic	PSF > 6 (*N* = 20)	PSF < 6 (*N* = 21)
Age		
< 50	4	4
> 50	16	17
Stage		
I-II	5	3
III-IV	15	18
Grade		
0	0	0
1	1	0
2	4	5
3	15	16
Histologic subtypes		
Serous	14	19
Mucinous	3	1
Endometrioid	3	1
Debulking status		
Optimal (≤1cm)	7	7
Suboptimal (>1cm)	13	14
Chemotherapy response		
Sensitive	20	0
Resistant	0	21

### Construction of Tet-On 3G inducible expression systems

Tet-On 3G inducible expression systems were purchased from Clontech Laboratories, Inc. The hsa-miR-497 and hsa-miR-NS fragments were PCR amplified from hsa-miR-497 and hsa-miR-NS overexpression plasmids obtained from Open biosystems. The purified fragments were inserted into pTRE3G plasmid to obtain pTRE3G-miR-497 or pTRE3G-miR-NS plasmid. pCMV-Tet3G plasmid was transfected into target cells, then selected by G418 for stably expressing cells. Then cells were transfected with pTRE3G-miR-497 or pTRE3G-miR-NS plasmid. After a second round of selection, cell lines were established and comfirmed for expressing high levels of hsa-miR-497 or hsa-miR-NS in response to doxycycline (Dox).

### Transfection of cells with pre-miRNA precursors or anti-miRNA inhibitors

Hsa-miR-NS, hsa-miR-497 precursors, or anti-miR-497 and control inhibitors (Ambion, TX, USA) were transfected into cells using Lipofectamine RNAiMAX (Invitrogen, CA, USA) according to the manufacturer's instructions. Protein lysates and total RNAs were collected 72 h after the transfection. The expression levels of miRNAs were verified by stem-loop qRT-PCR analysis.

### Quantitative real-time PCR

Total RNAs were isolated using Trizol Reagent (Life Technologies, Carlsbad, CA, USA) according to the manufacturer's instructions. cDNAs were synthesized with TaqMan Reverse Transcription Reagents (Life Technologies). The expression levels of miRNAs were analyzed using Taqman MicroRNA Assay Kits (Applied Biosystems, Foster City, CA) specific for hsa-Let-7e, hsa-Let-7i, hsa-miR-214, hsa-miR-497 precursors, p70S6K1 and mTOR mRNAs. The fold stimulation was determined using the comparative cycle threshold method (2^−ΔΔCT^) [37]. All experiments were performed in triplicate.

### Western blotting analysis

Cells were lysed in RIPA buffer with protein inhibitors. Total proteins (20-40 μg) from each sample were electrophoresised on 8 % SDS-PAGE gel, and transferred to a nitrocellulose membrane. The membranes were blocked in 5% nonfat milk and probed with the primary antibodies as indicated overnight at 4°C. The membranes were washed and probed with the secondary antibody conjugated to horseradish peroxidase and the developed with enhanced chemiluminescence (Thermo Scientific).R

### DNA methylation analysis

Genomic DNAs were purified using DNA Mini kit (Qiagen, MD, USA). Genomic DNAs were modified with sodium bisulfite using the EpiTect Kit (Qiagen) following the manufacturer's instruction, then analyzed by methylation-specific PCR (MSP) using primers for either methylated or unmethylated DNA.

Methylation-specific PCR primers are summarized as follows:

MiR-497 MF: 5′-TTGATTTAGGGAGAGGAAGGAC-3′

MiR-497 MR: 5′-TAAACAAACAACTAAAAAACGACGA-3′

MiR-497 UF: 5′-TTTGATTTAGGGAGAGGAAGGAT-3′

MiR-497 UR: 5′-AAACAAACAACTAAAAAACAACAAA-3′

### Luciferase reporter assay

mTOR and p70S6K1 3′-UTR regions containing predicted miR-497 binding sites and corresponding mutant sites were amplified by PCR from genomic DNA, and the PCR fragments were inserted into untranslated region (UTR) downstream of the luciferase gene in the pMIR-reporter luciferase vector (Ambion). Luciferase reporter plasmid, β-galactosidase (β-gal) plasmid, and pre-miR-497 and negative control precursors were cotransfected into cells using Lipofectamine 2000 (Invitrogen). Luciferase activities were measured 48 hours after transfection using β-gal for normalization. Primers used for Luciferase reporter constructs as follows:

mTOR Wild-type F: 5′-GCCGAGCTCTTTTCTGAGGCTTTTGTA-3′

mTOR Wild-type R: 5′-GCGAAGCTTCTAGGTCATTCTTCCATC-3′

mTOR Mutant F: 5′-GCCGAGCTCGGTTTGAACCAACTTTCTAGCTGCTGTTGAAGAATATATTGTCAGAAGCTTCGC-3′

mTOR Mutant R: 5′-GCGAAGCTTCTGACAATATATTCTTCAACAGCAGCTAGAAAGTTGGTTCAAACCGAGCTCGGC-3′

p70S6K1 Wild-type F: 5′-GCCGAGCTCTAGCCCTTGAGCCCTGTCC-3′

p70S6K1 Wild-type R: 5′-GCGAAGCTTATTCAGCCCTTTAATCTTCCAC-3′

p70S6K1 Mutant F: 5′-GCCGAGCTCGGAGATAGGGATATCCAGGGGAAGAGGGTGTAGCTGTGGCCCACAAGCTTCGC-3′

p70S6K1 Mutant R: 5′-GCGAAGCTTGTGGGCCACAGCTACACCCTCTTCCCCTGGATATCCCTATCTCCGAGCTCGGC-3′

### MTT assay

Cells (1×10^4^) were seeded into 96-well plates. After cellular adhesion, medium containing cisplatin at distinct concentrations (0.1-1000 μM) was added to the corresponding cells. After 72 h, cell viability was assessed using the MTT Cell Proliferation Assay (life technologies). MTT reagent was prepared in fresh medium (100 μL medium + 20 μL MTT solution) and applied to the cells. The absorbance at 590 nm for each well was read on a spectrophotometer [38].

### Mouse xenograft models

BALB/c athymic nude mice were housed and maintained in a laminar airflow cabinet in a pathogen-free environment. Mice were anaesthetized and ovarian were exposed. A2780/CP-Tet-On-miR-NS or A2780/CP-Tet-On-miR-497 cells (1×10^6^) were injected into the ovarian capsule via sterile microsyringe. The mice were feed drinking water with Dox to induce miR-497 expression 6 days after the injection. On day 4 after tumor cells implantation, cisplatin intravenously (i.v) by tail vein injections every 5 days. The mice were sacrificed by euthanasia. The maximum tumor size was not exceed 1 cm^3^. Primary tumors were harvested from mice, and the tumor volumes were determined according to the formula of 1/2 (length×width×height).

### Coexpression analysis of 2011 TCGA dataset

A normalized mRNA expression dataset for ovarian cancer [39] was downloaded from the cBioPortal for cancer genomics and used to evaluate coexpression of mTOR, p70S6K1, and miR-497 transcript levels. This dataset includes mRNA profiles for 489 primary tumor samples. Spearman's correlation coefficiency was calculated for these transcripts for all primary tumor samples. Differences were considered significant with *p* < 0.05.

### Statistical analysis

All results were analyzed using SPSS for windows version 13 (SPSS, Chicago, IL, USA). Quantitative variables were analyzed by *t*-test or ANOVA. The correlations were analyzed using Spearman's rank test. Differences were considered significant with *p* less than 0.05. Data represent mean plus SD from at least 3 replications, unless indicated specifically otherwise.
